# Digitalizing disease surveillance: experience from Sierra Leone

**DOI:** 10.1093/heapol/czae039

**Published:** 2024-05-30

**Authors:** Magoba Bridget, Gebrekrstos Negash Gebru, George S Odongo, Calle Hedberg, Adel Hussein Elduma, Joseph Sam Kanu, James Bangura, James Sylvester Squire, Monique A Foster

**Affiliations:** Department of Surveillance, African Field Epidemiology Network, Freetown, Sierra Leone; Department of Surveillance, African Field Epidemiology Network, Freetown, Sierra Leone; Sierra Leone Field Epidemiology Training Program, Freetown, Sierra Leone; Division of Global Health Protection, United States Centers for Disease Control and Prevention, 1600 Clifton Road, Atlanta, GA, United States; Health Information Systems Program, PostNet Suite, #47 Private Bag, X3 Beacon Bay, Pretoria, South Africa; Department of Surveillance, African Field Epidemiology Network, Freetown, Sierra Leone; National Disease Surveillance Program, Ministry of Health, Freetown, Sierra Leone; Department of Community Health, College of Medicine and Allied Health Sciences, University of Sierra Leone, Freetown, Sierra Leone; Health and Development in Action (HEADA), 16 Lower Pipeline, off Wilkinson road, Freetown, Sierra Leone; National Disease Surveillance Program, Ministry of Health, Freetown, Sierra Leone; Department of Community Health, College of Medicine and Allied Health Sciences, University of Sierra Leone, Freetown, Sierra Leone; Division of Global Health Protection, United States Centers for Disease Control and Prevention, 1600 Clifton Road, Atlanta, GA, United States

**Keywords:** Surveillance, DHIS2, eIDSR, eCBDS, Sierra Leone

## Abstract

The Integrated Disease Surveillance and Response (IDSR) system was adopted by the Sierra Leone Ministry of Health (MOH) in 2008, which was based on paper-based tools for health data recording and reporting from health facilities to the national level. The Sierra Leone MoH introduced the implementation of electronic case-based disease surveillance reporting of immediately notifiable diseases. This study aims to document and describe the experience of Sierra Leone in transforming her paper-based disease surveillance system into an electronic disease surveillance system. Retrospective mixed methods of qualitative and quantitative data were reviewed. Qualitative data were collected by reviewing surveillance technical reports, epidemiological bulletins, COVID-19, IDSR technical guidelines, Digital Health strategy and DHIS2 documentation. Content and thematic data analyses were performed for the qualitative data, while Microsoft Excel and DHIS2 platform were used for the quantitative data analysis to document the experience of Sierra Leone in digitalizing its disease surveillance system. In the early 2017, a web-based electronic Case-Based Disease Surveillance (eCBDS) for real-time reporting of immediately notifiable diseases and health threats was piloted using the District Health Information System 2 (DHIS2) software. The eCBDS integrates case profile, laboratory, and final outcome data. All captured data and information are immediately accessible to users with the required credentials. The system can be accessed via a browser or an Android DHIS2 application. By 2021, there was a significant increase in the proportion of immediately notifiable cases reported through the facility-level electronic platform, and more than 80% of the cases reported through the weekly surveillance platform had case-based data in eCBDS. Case-based data from the platform are analysed and disseminated to stakeholders for public health decision-making. Several outbreaks of Lassa fever, Measles, vaccine-derived Polio and Anthrax have been tracked in real-time through the eCBDS.

Key messages

- Before 2016, Sierra Leone had a paper-based disease surveillance reporting system characterized by untimely, incomplete, inaccurate and/or duplicated data. What this study adds: in the early 2017, District Health Information System 2 (DHIS2) software was customized for electronic Case-Based Disease Surveillance (eCBDS) for real-time reporting of immediately notifiable diseases.
- How this study might affect research, practice or policy: this paper provides the experience of Sierra Leone in implementing the digital diseases surveillance system, documents lessons learned and challenges faced during the implementation process, which can inform other similar settings in Africa and beyond to replicate in designing and implementing their surveillance systems.
- This paper describes implementing the electronic case-based disease surveillance system in Sierra Leone. By 2022, the country had implemented the electronic system across 95% of public health facilities, and more than 80% of the cases reported have case-based data. Several recent outbreaks, including Lassa fever, measles, vaccine-derived Poliovirus and anthrax, have been tracked in real-time through the eCBDS, enabling Ministry of Health (MoH) rapid responses and thus quicker suppression of those outbreaks.

## Introduction

Effective disease surveillance and outbreak response rely on timely, accurate and sufficient data for public health decision-making. In 1998, the Regional Office for Africa of the World Health Organization (WHO-AFRO) developed and introduced the Integrated Disease Surveillance and Response (IDSR) framework, a strategy for conducting comprehensive disease surveillance in the African region (Njuguna *et al*., 2019). Sierra Leone adopted IDSR in 2008, initially focusing on 36 communicable and non-communicable diseases that are characterized as either epidemic-prone or diseases targeted for eradication and elimination (Ministry of Health and Sanitation, S.L. *et al*., 2008). In 2015, the country revised the list to 21 priority diseases for immediate reporting that includes 12 epidemic prone diseases and 9 diseases targeted for eradication or elimination (Leone *et al*., 2015). The list has had several smaller revisions since 2015.

**Figure 1.**
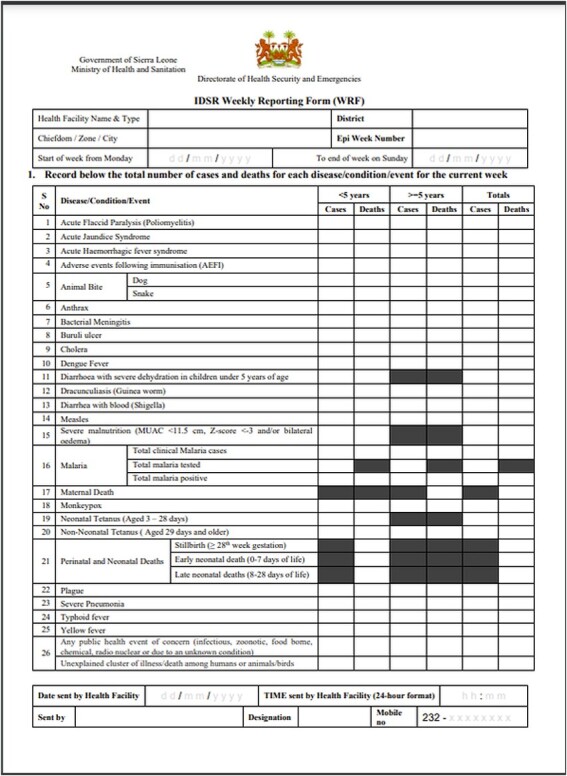
IDSR weekly reporting form, version 2021

Public health facilities started submitting weekly summaries of priority diseases to the district and national levels using a standardized paper reporting form (Figure 1, 2021 version). These reports were initially physically transported from the health facilities to the district offices to the National Directorate for Health Security and Emergencies (DHSE), and then increasingly captured into Microsoft Excel at the district level with those Excel files collated into one national file. This system was characterized by delayed and or incomplete reports. Accordingly, early detection of outbreaks can be achieved by timely and complete receipt, review and investigation of disease case reports (Buehler *et al*., 2004).

**Figure 2.**
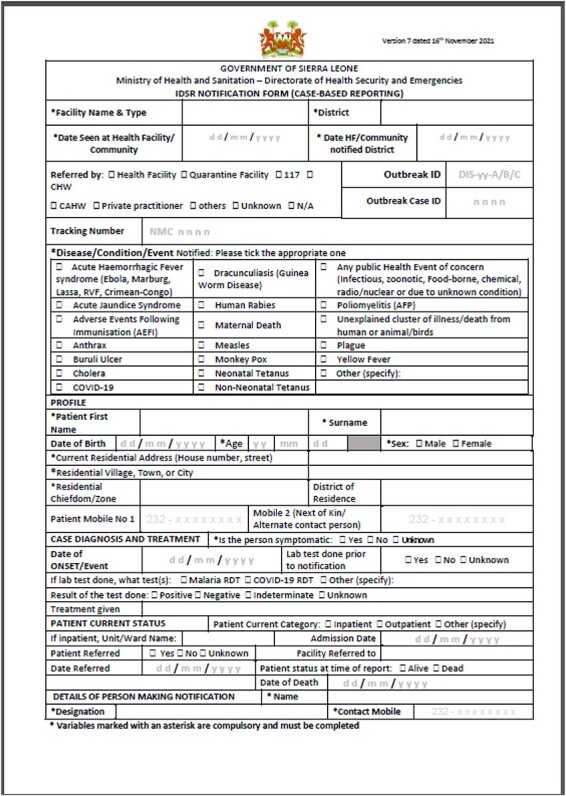
Case-based paper notification form, version 2021

The Ebola pandemic in West Africa in 2013–16 was a major warning both globally and in the countries affected—health-wise, socially, economically and culturally. Major gaps existed in data collection and transmission from one level to another due to lack of reporting tools and limited access to laboratory diagnosis of infectious diseases (Zhao *et al*., 2021). It also radically increased donor funding and international technical support towards disease surveillance—Government of Sierra Leone, WHO and United States Centers for Disease Control and Prevention (US CDC) expanded their activities. According to the Performance of Routine Information System Management Series (PRISM) framework (Aqil *et al*., 2009), health information systems are affected by the technical, behavioural and organizational determinants; the Ministry of Health (MoH) realized in 2015–16 the need for not only using digital tools for weekly data collection and analysis of suspected cases, but also the need for case-based reporting with laboratory results, case investigations, management of severe cases and final outcomes (Njuguna *et al*., 2019). The organizational determinants such as ownership and buy-in affects the information systems performance, support services, resources and organizational culture towards change from paper-based to electronic reporting (Aqil *et al*., 2009). Some health facilities were filling in the case-based notification form (Figure 2, 2021 version), but the information remained on paper which was not usable. The PRISM framework shows that the use of information is an output and data quality indicators such as completeness and timeliness are used to assess the processes of data collection and transmission District Health Management Team (DHMT) would be notified by a phone call if a suspected case of a priority disease was detected at a health facility. There was a mix of older and newer case investigation forms (CIFs) for some of the notifiable conditions, but any case investigations done also ended with the data remaining on paper in the district offices. Laboratory results were often late and/or did not reach district surveillance staff and facility clinicians at all. Additionally, user’s demand, confidence and motivation for data increase the system utilization (Aqil *et al*., 2009); with availability and access to laboratory results to clinicians and district surveillance teams, their confidence in the system utility and usefulness is improved.

Two development projects emanated from these developments, both supported by CDC and WHO: (1) facility-based reporting of weekly aggregated data of suspected cases for ∼27 notifiable medical conditions; and (2) facility-based case-based reporting with laboratory data, case investigation data, severe case management data and final outcomes.

## Methods

Retrospective mixed methods of qualitative and quantitative data were reviewed including the use of DHIS2 systems. Qualitative data were collected by reviewing surveillance technical reports, epidemiological bulletins, COVID-19, IDSR technical guidelines, Digital Health strategy and DHIS2 documentation. Content and thematic data analyses were performed for the qualitative data, while Microsoft Excel and DHIS2 platform were used for the quantitative data analysis to document the experience of Sierra Leone in digitalizing its disease surveillance system.

**Figure 3.**
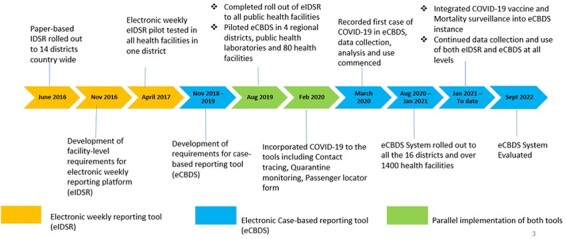
Electronic surveillance systems implementation timelines in Sierra Leone

Figure 3 shows the timelines and key milestones towards implementation of the electronic reporting systems: eIDSR and eCBDS.

### eIDSR reporting system project 2016–23

The electronic weekly reporting tool commonly referred to as electronic IDSR (eIDSR) is built using the District Health Information Software 2 (DHIS2), a free and open-source platform used by over 70 countries and many international and global organizations. Sierra Leone started using DHIS2 in 2008–2009 as a health management information system (HMIS) for routine monthly reporting of health indicators (Nielsen, 2012). Aggregated weekly disease surveillance data were added to the HMIS as a weekly ‘data set’ in 2016 and followed up with several weekly data collection form revisions, adding new disease conditions/events until their completion in 2019. This project was technically implemented by eHealth Africa (https://www.ehealthafrica.org/).

### eCBDS reporting system project 2018

**Figure 4.**
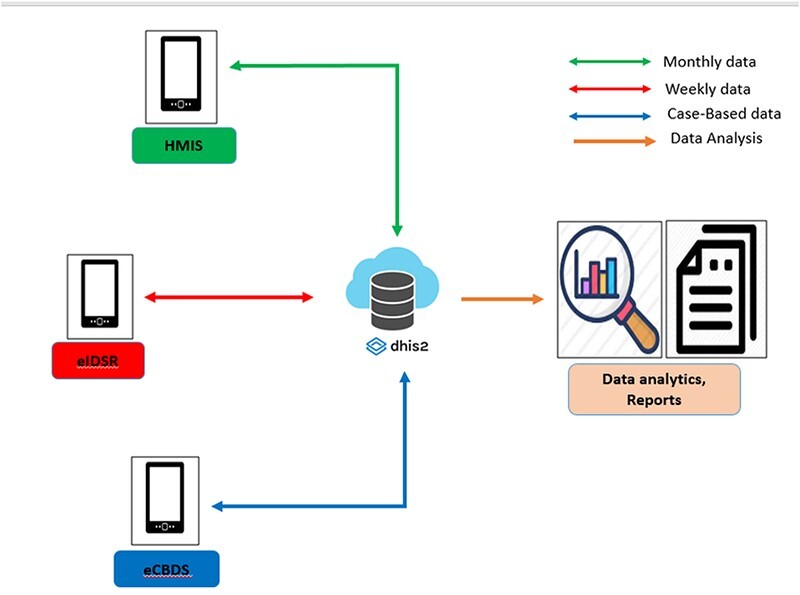
The relationship between HMIS, eIDSR and eCBDS data systems in Sierra Leone

In 2016, the MoH implemented the first electronic case-based disease surveillance reporting of around 20 notifiable diseases by email, SMS, radio or phone calls. In the early 2018, the second development project started with DHSE and the authors’ institutes establishing the electronic Case Base Disease Surveillance System (eCBDS). The eCBDS was configured as a new database on the DHIS2 platform using its tracker capture functionality, based on a template case-based IDSR design from South Africa and later revised and expanded numerous times. The eCBDS was configured on a separate DHIS2 instance from eIDSR which is configured on the HMIS. Core resources like the administrative/organizational hierarchy are fully aligned between the HMIS and the eCBDS instances. Figure 4 shows the relationship between the HMIS, eIDSR and eCBDS data systems in Sierra Leone. During 2018–19, it went through several pilot phases, with the author’s institute providing on-the-ground technical support during the adaptation of the IDSR third edition technical guidelines. New or modified versions of the case notification form, the disease-specific CIFs, the laboratory forms and the final outcome form were developed and distributed, combined with training of district and facility personnel in the use of both the paper forms and their eCBDS electronic equivalent. The eCBDS project has been built on the technical infrastructure (devices, SMS alerts) established by the eIDSR project, and both technical training and support to the districts and facilities are today well integrated. This integrated approach will be followed when facility-based data capture is expanded to include monthly aggregated data during 2023–24.

The COVID-19 pandemic fast-tracked the rollout of the eCBDS to the whole country in 2020 and was augmented to cover all important notifiable diseases in 2021.

A description of the technical working of the eCBDS is explained in the next section.

### Describing the eCBDS design

**Figure 5.**
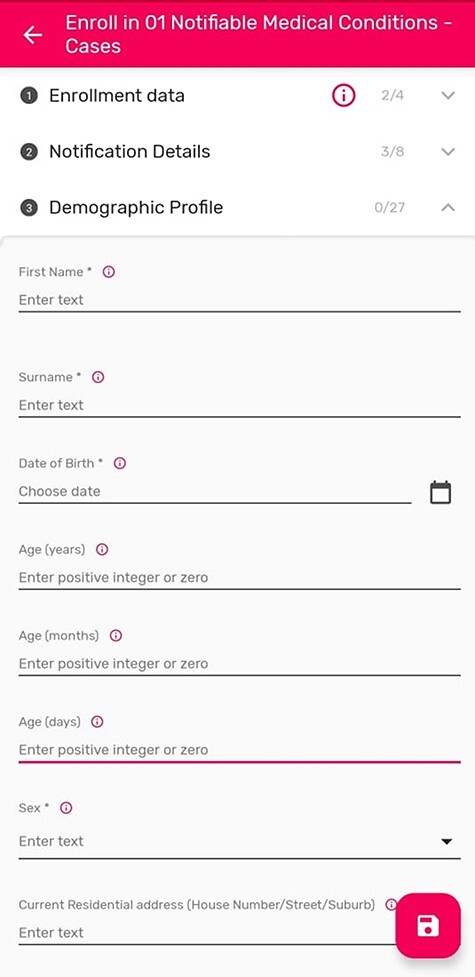
Electronic case-based notification form on an android tablet

The eCBDS is an effective surveillance electronic tool used for capturing and submitting individual-level data on priority diseases in real-time and designed using the DHIS2 Tracker Module (Health Information Systems Program, 2018; HISP Centre, University of Oslo (UiO), 2018). The data flow in the revised national IDSR technical guidelines was adapted to implement the eCBDS countrywide (Njuguna *et al*., 2019). When health facility staff detects a suspected case of an immediately reportable disease, a case-based notification form (Figure 2) is filled, and then the information is transformed from the paper form to an electronic version (Figure 5) using the tablet or a browser for the larger facilities that have laptops.

**Figure 6.**
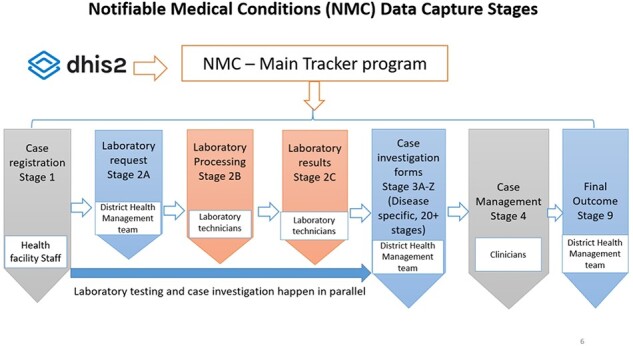
Electronic case-based disease surveillance system architectural design

**Figure 7.**
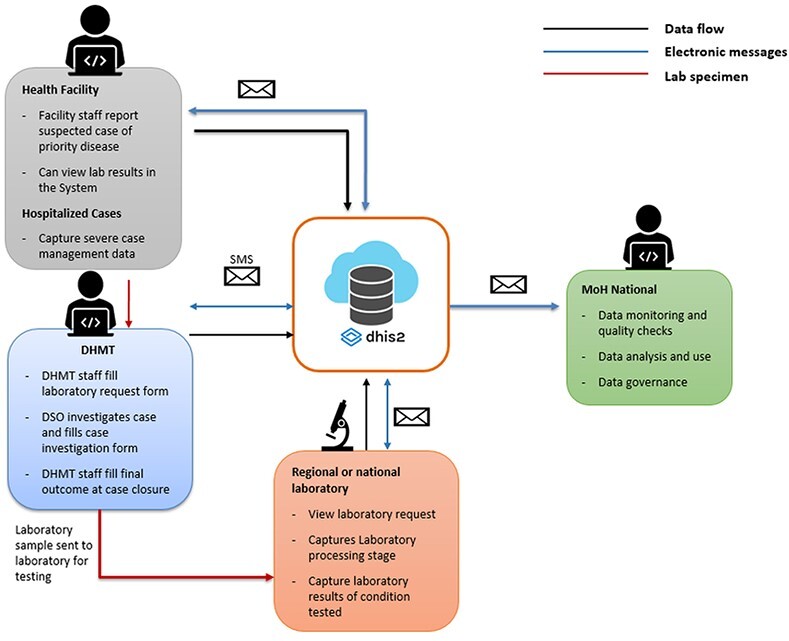
Electronic case-based disease surveillance system data flow diagram

The eCBDS has greatly improved the rapid bidirectional information sharing between the public health hierarchies of the country. Figures 6 and 7 show the eCBDS design and data flow, respectively, which are interlinked. When a health facility staff registers a suspected case of a priority disease into eCBDS at Stage 1, the system auto generates a unique patient identifier called ‘Notifiable Medical Condition Case Identifier’ (NMC CaseID) which is used to uniquely identify cases reported through eCBDS by national, district and laboratory level. The system immediately sends a notification (SMS and/or email) as shown in Figure 6 to the relevant user group. This triggers a case investigation by district surveillance officials at Stage 3A-Z based on the disease reported. The system will display the CIF of the disease reported at Stage 1. Meanwhile, during investigation, if a specimen is required, the district laboratory technician collects the specimen and captures a laboratory request at Stage 2A. A notification message with minimal detail is sent to the reference laboratory where the specimen is referred as shown in Figure 7 of lab specimen movement from health facility to district and finally to the regional or national laboratory. This process is followed by the laboratory processing information on receipt of the specimen at Stage 2B (Figure 6) captured by the laboratory technician. Similarly, upon completion of specimen analysis, the laboratory technician captures laboratory results into the eCBDS at Stage 2C which triggers a notification immediately to the national surveillance officials showing that ‘the laboratory results are ready’. District officials could also be notified immediately, but currently this is prevented because of the patient laboratory result confidentiality and therefore limited to the head clinician. The clinician at the health facility views laboratory results through eCBDS but does not have write access to Stage 2C. As the case investigation is happening in parallel with the laboratory processes by the district surveillance team, the clinicians at the health facility capture data on case management for the hospitalized severe cases at Stage 4 and finally, the surveillance officers capture the final outcome of the case on closure at Stage 9. The national staff monitor all the data captured through the system across all stages, and run data quality checks through routine data analysis and quarterly data quality assessment at district and health facility levels. The system is set up with DHIS2 programme rules for data quality checks. Currently, the MoH has established several strategies for surveillance data use at all levels since the roll out of the electronic systems. Technical working groups composed of MoH staff and partners have been set up to discuss technical matters based on data from lower levels of reporting, the impact and public health actions to be taken. Similarly, the disease-specific data extracted from the system and monitored on the dashboard are analysed to assess disease trends, mortality rates, geographical coverage among other indicators to make public health decisions.

## Results

### The expansion from eIDSR to electronic case-based disease surveillance reporting (eCBDS)

The implementation of eCBDS was conducted in a phased and coordinated approach. Four out of 16 districts based on regional representation and the success of eIDSR were selected to pilot the use of eCBDS between 2018 and 2019. Initial implementation focused on using the system at the district level to ride on existing infrastructure and minimize costs (Sloan *et al*., 2020). During this pilot phase, capacity building of 40 staff from DHMTs, national surveillance office and implementing partners were achieved through training, on-the-job mentorship and routine site support visits.

### Capacity building

After 7 months of pilot and capacity building at the district level, eCBDS was rolled out to 80 health facilities in the 4 pilot districts and 6 regional referral laboratories in mid-2019. The roll out was extended to the health facility level because it is the first point of contact for a case; the district health teams were overwhelmed with other district-level activities and programmes. The piloting of eCBDS at health facilities was made possible by the use of the already existing tablets at health facilities and provision of data for internet connection. Training of health facility and regional laboratory staff was conducted by joint national and district teams. On-site and on-the-job mentorship continued throughout this pilot phase. National technical teams routinely monitored the performance of the system at health facilities. Informal assessments were conducted in the four pilot districts through a review meeting with the key district and health facility stakeholders. The objectives of the review meeting were to assess output of eCBDS from health facilities, share experience, challenges and lessons learnt and plan a way forward on the national roll out. Data from eCBDS showed that 60 (75%) out of the 80 health facility trained staff had reported at least one priority disease through the system, and 45 (75%) of the 60 cases were investigated. The successful implementation at the health facility level informed the decision to roll out the system to all public health facilities throughout the country.

A three-tiered strategy was adopted to fast-track the rollout of eCBDS nationally. Training of national trainers (ToT) was conducted to cascade training to lower levels. The national training targeted Information Technology (IT) specialists, data managers and officers, surveillance officers, laboratory data officers and technicians, who were trained on data capture and analysis using DHIS2 tracker capture and android application. The data capture span from notification to all stages; notification, lab request, lab processing and results for the reference laboratory officers, case investigation using the web and android applications. The national trainers’ data analysis content included intermediate analysis by line list, pivot tables, charts and graphs, epi-curve, maps with an epidemiological perspective using the web application. In addition, the national trainers received training on data visualization using DHIS2 dashboards, creating them and adding analysis items based on need. In March 2020, eCBDS training was cascaded to the remaining 12 districts alongside the rollout of technical guidelines for IDSR third edition (Ministry of Health and Sanitation, 2020). National trainers were instrumental in conducting training for district staff at regional level targeting district surveillance officers, data officers, district and hospital laboratory technicians. The training content for district staff was limited to filling the notification using web and android application on the tablet and case investigation hard copy forms using real-life scenarios, which was then captured in the electronic system (eCBDS) at the case investigation stage, capturing laboratory request stage upon specimen collection and final outcome of the case using the web. The district-level staff were also trained on basic data analysis and visualization using DHIS2. Data analysis tools focus on generating a line list, pivot tables, graphs and charts, maps and visualizing the data on the dashboard. The health facility training was conducted at the district level targeting facility in charges or IDSR focal persons, facilitated by DHMT staff supported by national trainers and partner staff. The health facility training content included basic use of tablets, filling the notification hard copy using a real-life case scenario and data capture into eCBDS using a DHIS2 android capture application. Initially, the application did not have the data analysis functionality until September 2023 when the country introduced analytics on the tablets at health facility level. This training built and enhanced the capacity of 1469 staff from 1419 public health facilities and 90 DHMT staff. Training in eCBDS use was also extended to over 100 private health facilities staff across the country. The dedicated technical team composed of one systems administrator who manages the server administration and data security (mainly the system backend) with strong computing and programming skills, two systems analyst designs, configures the system as requirements change with skills in computing and DHIS2 and an epidemiologist or health informatics specialist with public health and computer background to give direction on epidemiology. The team at national level is responsible for system maintenance, system or functionality upgrades and advanced technical support to users among others.

### Case notification, detection and investigation (data)

**Figure 8.**
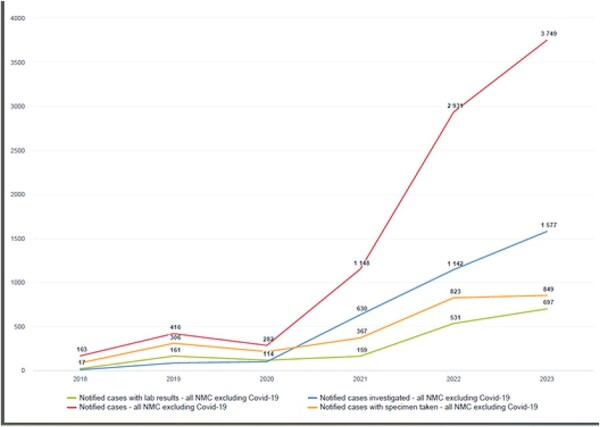
A line graph showing the progress of eCBDS use over 6 years in Sierra Leone

The data in Figure 8 cover all the immediately reportable priority diseases but deliberately exclude COVID-19 cases because the system mainly captured confirmed cases. In 2018, only 163 cases were notified before the full implementation of eCBDS; there were no case investigations recorded that year. In 2019, during the eCBDS pilot, the cases increased to 416 with 73.5% of cases with specimen taken, 52.6% had lab results captured in the system. The case investigations have been inconstant over the years from 54.8% of cases investigated in 2021 to 42% of cases investigated in 2023.

In 2020, 54% of the lab results of the specimens sent to the public health laboratories were captured in the system compared with 43% captured in 2021, as shown in Table 1. However, there was increase in system use by the laboratory staff by 2022 where 65% of specimens had results captured, increasingly in 2023.

**Table 1.** Shows laboratory data completeness across 4 years

Reporting year	Notifiable cases—all NMC excluding Covid-19	Notifiable cases with specimen taken—all NMC excluding Covid-19	Notifiable cases with lab results—All NMC excluding Covid-19	Percentage of specimen with lab results captured in eCBDS
2020	282	211	114	54
2021	1148	367	159	43
2022	2930	822	531	65
2023	3751	853	697	82

Table 2 shows a comparison of the weekly aggregate reporting—highlighted in blue—to the case-based reporting—highlighted in orange—over 4 years for five immediately notifiable diseases/conditions: Acute Viral Haemorrhagic Fever (AVHF), Acute Flaccid Paralysis (AFP), Measles, Neonatal tetanus (NNT) and Yellow fever. In 2020, an average gap of 36 cases of the suspected cases reported in eIDSR were not reported in eCBDS. However, in 2021, 168 AFP cases were reported in eCBDS as compared with 158 cases in the eIDSR. By 2022, both AFP and AVHF had a gap of six and five cases reported in eCBDS as compared with eIDSR. In 2023, all the 5 diseases had an average gap of 62 cases reported in eCBDS as compared with eIDSR.

**Table 2.** Showing data comparison between eIDSR and eCBDS across 4 years

Reporting year	2023	2022	2021	2020
eIDSR—Acute Flaccid Paralysis	167	138	158	118
eCBDS—Acute Flaccid Paralysis	204	144	168	51
Gap	37	6	10	−67
eIDSR—Acute Viral Haemorrhagic Fever	225	139	139	118
eCBDS—Acute Viral Haemorrhagic Fever	290	144	110	153
Gap	65	5	−29	35
eIDSR—Measles	1144	1179	152	112
eCBDS—Measles	1299	1073	149	18
Gap	155	−106	−3	−94
eIDSR—Neonatal tetanus	29	85	60	23
eCBDS—Neonatal tetanus	22	35	17	4
Gap	−7	−50	−43	−19
eIDSR—Yellow Fever	146	93	62	50
eCBDS—Yellow Fever	208	89	45	16
Gap	62	−4	−17	−34
**Average gap**	62	−30	−16	−36

## Discussion

This manuscript described the implementation of the electronic case-based disease surveillance system in Sierra Leone. By 2022, the country had implemented the electronic system across 95% of public health facilities in 16 districts. There is improved quality of case-based surveillance data reported from the health facility level, as a case is notified to the district level through eCBDS within an average of 12 hours with 100% mandatory variables captured. Data completeness, timeliness and availability for use at all levels have been greatly improved. The system has shown tremendous usefulness for early detection and response to disease outbreaks in the communities (Youssef *et al*., 2022).

The eCBDS system has improved case notification, detection from health facility level and investigation at district level over the past 6 years. In 2020, there was a reduction in case notification as the country focused on COVID-19 pandemic. However, the system picked up in 2021 as COVID-19 was introduced as a routine disease for monitoring. The case investigations have been unstable over the years due to the huge number of suspected cases reported especially the dog and snake bites; similarly, there are disease conditions and events added to the list of immediately reportable priorities during the adaptation of the IDSR third edition (Ministry of Health, Sierra Leone, 2020).

Stakeholder engagement increases awareness, fostering trust and confidence and buy-in of the system (Sedmak, 2021), even if it might slow down some processes to ensure all key stakeholders are on board. Laboratories play a critical role in the diagnosis of priority diseases and thus early detection and the need for linkage to surveillance (Onyebujoh *et al*., 2016), and there were lengthy discussions and several failed efforts before they came on board fully in mid-22. During 2020–22, several attempts to get laboratory data captured timeously failed because permanent staff in the ∼20 district and regional laboratories resisted changing from paper and Excel to an online system. This challenge was addressed through institutional arrangements such as recruitment, training and mentoring of full time data officers for the six public health laboratories. These officers were incentivized through resources from development partners.

By the end of 2022, case-based and weekly aggregated data reporting were at the same level—with a high proportion of eCBDS cases also having laboratory data and case investigation data. Disease surveillance personnel are increasingly using and relying on case-based data more than aggregated data because it is more real-time and provides a richer picture with lab confirmation and case investigation data that enables a more focused response to real threats. For example, eCBDS collects demographic, clinical and exposure history variables which are not captured in aggregated data collection approach. For a single measles case, demographic variables such as age, sex, residential address and clinical variables such as clinical signs and symptoms, immunization status as well as exposure status such as travel history linked with laboratory processes are collected through eCBDS while aggregate data collect only grouped variables (e.g. under 5 years and above 5 years). This gradual shift is expected to continue.

The electronic data collection tools initially designed aligned with the already existing paper-based tools used in the field to reduce confusion among the surveillance field staff and ease their transition to electronic reporting. Many of these collection tools were later modified and modernized.

Sierra Leone has built capacity of staff from the national, district and health facility levels to manage and use the system effectively. Continuous training is paramount to the sustainability of the systems as well as increasing exposure and buy-in (Moucheraud *et al*., 2017). Integrating the electronic case-based disease surveillance system into the author’s institute curriculum increased system use and ownership. Currently, though, there is still significant reliance on funding partners to support the technical development and maintenance of health information.

Mobile Device Management (MDM) software is used to control, secure and enforce policies on smartphones, iPhone, tablets and other endpoints. The country purchased the Manage Engine Mobile Device Manager software and installed on tablets at the health facility level to limit misuse, enhance security of data, enable remote control on application upgrades, thus reducing travel expenses for the technical team at $12 495 per device 2500 devices and $595 for two technicians per annum (ManageEngine, 2001). Sharing experiences through conferences for learning has promoted the work in Sierra Leone. Experiences shared through DHIS2 conferences, Health Informatics forums and public health forums such as Training Programs in Epidemiology and Public Health Interventions Network (TEPHINET) have given other countries an opportunity to learn from Sierra Leone’s experience in electronic disease surveillance systems. Similarly, through these forums, Sierra Leone learns how to improve its implementation based on experiences shared by other countries.

Interoperability is the ability of different health information systems to work together within and across different pillars or departments. The eCBDS, as a highly integrated system for disease surveillance, will nevertheless need to be interoperable with, for instance, the system used by the animal disease sector (also known as the One Health approach), with Human Resource systems, laboratory systems and so on. The key principle we follow is to use integration where suitable, but also pursue interoperability for a reduction in bureaucracies leading to increased data use (George *et al*., 2020; Torab-Miandoab *et al*., 2023).

The DHSE conducts weekly multisectoral partner meetings to review and analyse data from different parts of the country for informed decision-making. The periodic data analysis increases data use at different levels of generation. The use of surveillance data promotes wider dissemination, improving the basis for evidence-based public health decisions. Data quality assessments are conducted quarterly to monitor gaps in reporting, data quality and an opportunity for on-the-job mentorship at the health facility level. However, the case-based hard copy reporting forms at the facility level were less utilized because facilities hardly detect and suspect priority diseases (Groseclose and Buckeridge, 2017).

Having the laboratories directly capturing lab results for priority diseases has greatly improved the turnaround time of laboratory results from over 5 days to 24 hours, improving feedback to the district and facility level as results are accessed through the electronic system (Onyebujoh *et al*., 2016). The implementation and success of eCBDS increased the country’s Joint External Evaluation (JEE) surveillance scores from Level 3 (Developed capacity) in 2018 to Level 4 (demonstrated capacity) in 2021 for the two indicators of the use of electronic tools and analysis of surveillance data (Ministry of Health, Sierra Leone, 2021). This helps the country in adhering to the International Health Regulations (IHR) requirements of early detection, prevention and control of priority diseases. The DHSE, responsible for disease surveillance, created an IDSR focal person role at the health facility level to take on surveillance responsibilities, including detection and reporting of any suspected cases. This role is performed for both the weekly and case-based reporting in all health facilities. This was not a new position, but an added role for existing health facility staff. At the district level, there are now rapid response teams at the DHMT that respond to any threats in their districts at the community or health facility level. The national level has set up a routine emergency preparedness and response technical working group responsible for reviewing data and making public health decisions.

However, there are gaps identified in the surveillance system that need to be addressed, including staff attrition, obsolete devices for reporting, new parallel applications developed, over-dependence on development partners and infrastructural challenges. Trained and skilled health facility staff are often transferred to other programmes or health facilities creating gaps in their previous facilities on the use of eCBDS for reporting due to knowledge gap. There is limited transfer of knowledge by the health facility staff to their fellows.

The functionality of the mobile device tablets is key in maintaining the electronic system. The country has faced a challenge of obsolete and outdated tablets with limitations including breakdown and/or overheating, incompatibility with DHIS2 due to low android version which hinder their use. Fortunately, the MoH has set minimum specifications for devices as clearly defined in the national digital health roadmap (Ministry of Health, Sierra Leone and Ministry of Information and Communication, Sierra Leone, 2023), and the ministry now has a reasonably up-to-date registry of all government devices. During the 2019–20 COVID-19 pandemic, there were a number of looming applications developed and presented to the response leadership for data collection in parallel with the existing eCBDS system, which caused confusion, double reporting and waste of resources. The parallel systems were not sustained when COVID-19 funding reduced, thus the country losing data. A number of the electronic implementation resources including the infrastructure, trainings and human resource depend on donor funds which are not sustainable.

The Ministry in collaboration with health development partners is working towards resolving the challenges affecting the effectiveness of the electronic surveillance system (Fall *et al*., 2019).

Other challenges include the development, setup, implementation and maintenance of the electronic surveillance system. Resources required to maintain an electronic system include stable internet infrastructure, usable electronic devices and data bundles or zero-rated internet access. Although the MOH has sustainable human resources capacity to maintain the system, it may depend on their partners to some extent for financial sustainability (Mandyata *et al*., 2017).

The reporting nature of the case-based system requires several stages and different individuals. Therefore, delays in registering cases affect the capture of laboratory results. The stages in the flow of information are dependent on each other (Reynolds *et al*., 2022).

## Conclusion

The Ebola outbreak in Sierra Leone between 2014 and 2016 exposed huge weaknesses in the surveillance system. The MoH has made significant strides to strengthen the surveillance system for early detection, prevention, control and reporting of priority diseases. So far, the Ministry has put in place a case-based surveillance system for 19 priority disease events and conditions, developed and rolled out to all public and 75% of private health facilities for early detection, prevention and reporting by end-2022. There is improvement in reporting, investigating and following up priority disease cases facilitated by the availability of a system for real-time reporting and data sharing. Suspected case reporting time has improved from 72 hours to at most 24 hours from the time a case is detected at a health facility. Throughout the COVID-19 pandemic, the country took the opportunity to utilize the same resources and roll out the system for both COVID-19 and other priority cases, contacts, quarantine, travellers and laboratory data collection. The pandemic promoted unity of efforts as part of a shared threat for the response and the routine surveillance. However, we also saw a number of standalone systems not integrated with the eCBDS, creating parallel reporting channels and causing confusion and extra work. Those parallel systems collapsed towards the end of the pandemic including Health Connect for Case management and Laboratory results capture and dissemination, Travel portal (Http://travel.gov.sl), Quarantine Monitoring tool using CommCare.

The future direction for the electronic surveillance system is to continue integrating new components or else to ensure interoperability with other systems. There are plans to build advanced technical capacity at the country level for sustainability and future maintenance of the system.

## Reflexivity Statement

The authors include two females and seven males and span multiple levels of seniority. Seven authors are located in Sierra Leone, West Africa, one author in US Atlanta and one in Norway. Three authors specialize in Health Information Systems while three are medical doctors with epidemiology background. All the nine authors have experience in conducting health policy analysis and conducting research.
